# Effects of a mindfulness-based health promotion program on mindfulness, psychological capital, compassion fatigue, and affect in healthcare workers

**DOI:** 10.3389/fpsyg.2024.1470695

**Published:** 2024-10-24

**Authors:** Eliana Quiroz-González, María Laura Lupano Perugini, Leonor Emilia Delgado-Abella, Jaime Arenas-Granada, Marcelo Demarzo

**Affiliations:** ^1^Program of Psychology, Universidad Católica de Pereira, Pereira, Colombia; ^2^National Scientific and Technical Research Council (CONICET), Buenos Aires, Argentina; ^3^Department of Psychology, Universidad de Palermo, Buenos Aires, Argentina; ^4^Faculty of Psychology, Universidad de Buenos Aires, Buenos Aires, Argentina; ^5^Faculty of Psychology, Universidad El Bosque, Bogotá, Colombia; ^6^Faculty of Physical Education, Recreation and Sport, Politécnico Colombiano Jaime Isaza Cadavid, Medellín, Colombia; ^7^Department of Preventive Medicine, Mente Aberta-Brazilian Center for Mindfulness and Health Promotion, Universidade Federal de São Paulo, São Paulo, Brazil

**Keywords:** affect, compassion fatigue, healthcare workers, mental health, mindfulness, psychological capital

## Abstract

**Objective:**

This study aimed to examine the effects of the Mindfulness-Based Health Promotion program on mindfulness, psychological capital, compassion fatigue, and affect in a sample of healthcare workers at a hospital in Colombia.

**Method:**

Randomized controlled study with pre-test and post-test measures, in which 33 workers participated (Mage = 35.01, SD = 10.0), assigned to the experimental group (EG, *n* = 16, Mage = 34.00, SD = 9.59) and the wait-list control group (CG, *n* = 17, Mage = 36.03, SD = 10.56). The contrast of the program effects was carried out through a mixed factorial ANOVA.

**Results:**

We found significant effects on observing, non-reacting and mindfulness as a function of the group factor.

**Conclusion:**

We concluded the Mindfulness-Based Health Promotion program showed positive effects on mindfulness, this was tested with a novel program designed for the Latin population. This study is pioneering in using this mindfulness program in Colombia. These findings offer valuable insights for leaders of healthcare institutions when developing intervention programs that promote the mental health in the workplace. Future studies with larger samples and mixed-methods are necessary to confirm our results and to identify factors that can predict these findings.

## 1 Introduction

The work environment is often prone to generate high-stress levels, representing detrimental consequences at physical, social, and psychological levels (Yaribeygi et al., 2017). Several systematic reviews and meta-analysis have shown that work stress has effects on decreased productivity (Roczniewska et al., 2022; Timotius and Octavius, 2022), increased absenteeism (Taibi et al., 2021), staff turnover (He et al., 2020; Yang et al., 2021), translating into economic losses to companies (Schmidt et al., 2019).

One group of workers who face challenging situations and often experience high levels of occupational stress is healthcare professionals, especially those working in hospital settings (Assis et al., 2015; Borhany et al., 2023). Thus, there is a marked need to generate interventions that can decrease stress and promote health in such employees (Janssen et al., 2020; de Carvalho et al., 2021).

In addition to the situation mentioned above, healthcare workers were exposed to unparalleled work and emotional overload due to the COVID-19 pandemic, which generated a global health crisis (Yao et al., 2020), affecting health systems and led to repercussions on mental health (Orrego, 2024) and human resources management processes (Presti and Mendes, 2023). The impact of the pandemic on the mental health of healthcare workers was initially reported in China, with high rates of depressive symptoms, anxiety, insomnia, and distress (Lai et al., 2020). Since then, similar findings have been reported in other countries and cultures (Vindegaard and Benros, 2020).

Against this backdrop of mental health challenges, over the past few years, research and popular interest in mindfulness practices have increased (Trombka et al., 2021; Davies et al., 2024; Hsieh and Li, 2024). Mindfulness is the awareness that arises from paying attention, with purpose, to the experience that emerges in the present moment, without judgment or criticism. Such a state of awareness can be enhanced by simple meditation practices and through different MBIs (Kabat-Zinn, 1982).

Jon Kabat-Zinn and his colleagues created the Mindfulness-Based Stress Reduction (MBSR) program in 1979. It was an intervention whose effects on mental health and quality of life have produced several studies worldwide, both in clinical and non-clinical populations. Additionally, several MBSR-based protocols were developed targeting specific populations, such as the Mindfulness-Based Health Promotion (MBHP) program (Demarzo and García-Campayo, 2015), employed in the present study. This mindfulness program was designed and implemented by Centro Mente Aberta in Brazil and the Zaragoza University in Spain. It is inspired by the original model of Jon Kabat-Zinn (MBSR) but adapted to the context of health promotion and quality of life. It also adopted ideas from other programs, including some used in British centers, such as Mindfulness-Based Cognitive Therapy (MBCT) and Mindfulness-Based Relapse Prevention (Demarzo and García-Campayo, 2015; Duran et al., 2022).

Several studies and reviews address the effects of Mindfulness-Based Interventions (MBI) applied to work contexts and, particularly, healthcare workers (Rodriguez-Vega et al., 2020; Kim et al., 2022; Di Mario et al., 2023; Lin et al., 2024; Ong et al., 2024). If we limit ourselves only to those focusing on healthcare personnel, in general, they reported a reduction in stress levels and an increase in perceived well-being, the latter understood as a construct that includes life satisfaction and positive affect (Lomas et al., 2019). Given the interest generated by the study of the efficacy of MBIs in healthcare personnel, several reviews analyzed their effects on specific professions within the medical-care field, including nursing personnel (Guillaumie et al., 2017); mental health professionals (Rudaz et al., 2017) and medical students (Daya and Hearn, 2018). Overall, the reviews consulted highlight a disparity in the quality and types of interventions conducted, as well as a greater need for controlled trials (Lomas et al., 2019).

It is possible to use the MBHP program in Colombia, as it was not only designed for Brazil, but also for the Hispanic and Latin American public in general. Moreover, its application covers different health, educational, and organizational areas. The application of this program had shown its efficacy and effectiveness, both nationally and internationally. It increases the quality of life and decreases anxiety, depression, and burnout symptoms, primarily through techniques such as decentering and self-compassion (Demarzo and García-Campayo, 2015; Demarzo, 2020). These results were reported in studies conducted with police officers (Trombka et al., 2021), patients with post-traumatic stress disorder (Duran et al., 2022), and female teachers (Wilson et al., 2022), among others.

As with the classic mindfulness approaches (MBSR and MBCT), the goal of MBHP is to develop awareness -full consciousness- through the practice of mindfulness (which involves attention, attitude, and intention). Awareness is understood as being conscious of (realizing, recognizing, perceiving, noticing, and observing) internal (thoughts, feelings, emotions, sensations, and impulses) and external (activities, relationships, etc.) phenomena. The premise (principle/intention) is that if individuals are aware, they are more likely to make more assertive/conscious decisions/choices and respond to situations less reactively. There is scientific evidence showing that the development of awareness is one of the basic mechanisms that explain the benefits of mindfulness in promoting health, as it fosters self-efficacy and quality of life (Duran et al., 2022).

### 1.1 The present study

Different reviews on MBHP show its impact on psychological well-being and reducing symptoms associated with stress. In this research, the effect of the application of MBHP on a group of workers in a health institution in Valle del Cauca, Colombia, was analyzed. The effect of the intervention was analyzed in terms of mindfulness, psychological capital, compassion fatigue, and positive/negative affect perceived by these workers compared to a wait-list control group of workers from the same institution who did not undergo MBHP.

In the present study, the Five Facet Mindfulness Questionnaire (FFMQ) was used, which includes five dimensions (Observing, Describing, Acting with Awareness, Non-judging, and Non-reacting) and allows us to measure the tendency to engage in mindfulness in daily life. Numerous studies have shown that mindfulness practice is associated with increased trait mindfulness (Goldberg et al., 2016; Quaglia et al., 2016). Additionally, previous research has indicated that the FFMQ is sensitive to intervention and shows differences between mindfulness practitioners and non-practitioners (Van Dam et al., 2009), although some studies found changes in active controls and wait-list controls (Tran et al., 2022). For this reason, it was important to include it in this study. When results of systematic reviews, including the application of MBI in health professionals, are analyzed, it is observed that effect sizes are larger when a total score in mindfulness measurements is considered (Lomas et al., 2019). Previous research using the FFMQ reports a more significant effect on Non-reactivity (Asuero et al., 2014). However, no generalized trend is observed in the results. Based on the above, the following hypothesis is presented:

*H1*: Participants who go through MBHP training will have an effect on mindfulness considered overall.

Psychological capital is defined as a state of positive psychological development of the human being. This construct has been identified as a second-order factor. First-order factors are hope, optimism, resilience, and self-efficacy (Avey et al., 2006). The study of the effect of mindfulness training on it is not yet sufficiently documented. However, some research on some occupational groups, such as leaders of public and private organizations (Biswal and Srivastava, 2022) had reported positive effects. In India, in a sample of 64 adults consisting of housewives, health professionals, and students, Jain and Singh (2016) delivered an MBSR program to the experimental group (32 people) and found significant improvement in hope, optimism, resilience, and self-efficacy.

Xu et al. (2021) concluded from a systematic review of effective virtual interventions on psychological capital that mindfulness-based programs exhibited increased psychological capital measures. However, Samouei and Ghasemi (2015), using basic mindfulness training with health science students, reported non-significant differences in psychological capital measures between the control group and the study group. Based on the above evidence, the following hypothesis is formulated:

*H2*: Participants who go through MBHP training will have a significant effect on psychological capital.

Regarding compassion fatigue, healthcare professionals are a population at risk for high levels of compassion fatigue. A systematic review revealed that MBSR interventions effectively maintain and increase levels of mindfulness and self-compassion and decrease burnout, depression, anxiety, and stress. Overall, mindfulness was found to be effective in reducing negative affect and compassion fatigue (Conversano et al., 2020). In the same direction, by applying MBSR programs to nursing staff, different findings indicate that mindfulness-based interventions can effectively reduce compassion fatigue in this occupational segment (Best et al., 2020; Owens et al., 2020).

Duran et al. (2022) found that online MBHP training effectively treated post-traumatic stress in a group of patients and healthcare professionals diagnosed with COVID-19 or who had been in isolation or quarantine. Based on the above empirical background, the following hypotheses are formulated:

*H3*: Participants who go through MBHP training will have a change in measures of compassion fatigue; an increase in compassion satisfaction and a decrease in secondary traumatic stress and burnout measures are expected.

Finally, in relation to positive/negative affect, the reviews consulted generally report disparity in the methods used to assess it. In general, meta-analyses on MBHP report a small but significant effect on the decrease of negative affect (NA) after the implementation of the practices (Schumer et al., 2018), even when these are applied through technological devices (Victorson et al., 2020); while some studies report an increase in positive affect (PA) after having applied some modality of MBHP (Strege et al., 2018). MBHP had been shown to be effective in both increasing PA and decreasing AN in samples of teachers (Rodrigues de Oliveira et al., 2021). Consequently, the following hypothesis is formulated:

*H4*: Participants who go through MBHP training will have an effect on measures of positive and negative affect; a decrease in negative affect and an increase in positive affect are expected.

Healthcare professionals require psychosocial support to promote their well-being (Stuijfzand et al., 2020). In Colombia, few studies test intervention programs aimed at preventing psychosocial risks or promoting psychological resources among workers. In this regard, the results of this research add evidence to the effectiveness of MBHP, while guiding the selection of healthy organizational practices in the healthcare sector. Based on the above, this study aimed to examine the effects of the mindfulness-based health promotion program on mindfulness, psychological capital, compassion fatigue, and affect in a sample of healthcare and administrative staff at a hospital in Valle del Cauca, Colombia.

## 2 Materials and methods

### 2.1 Participants

**Figure 1:**
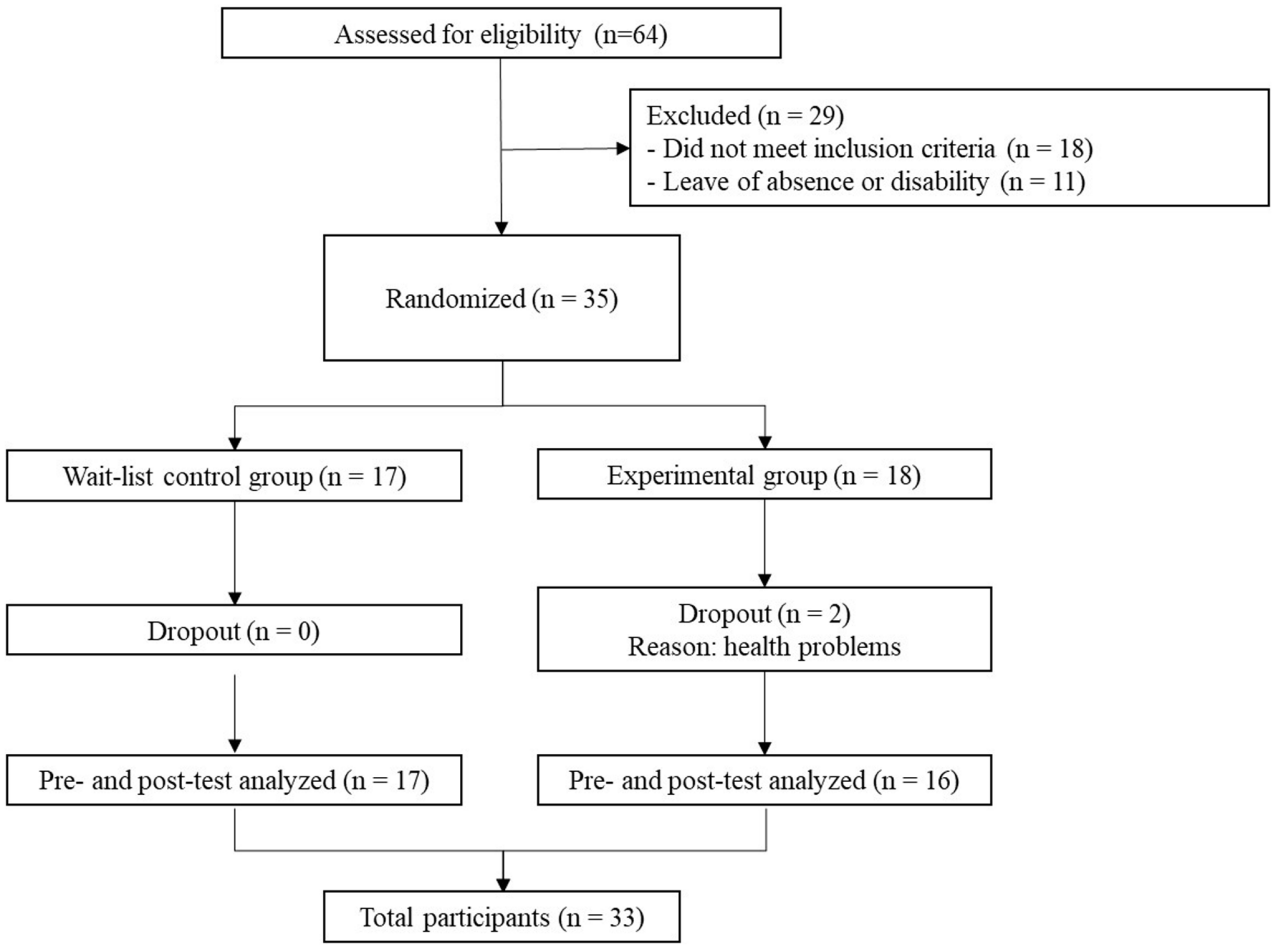
The flow of participants to take part in the Mindfulness-Based Health Promotion Program. *p < 0.05, **p < 0.01.

A randomized controlled experimental design with pre-test and post-test measures was used. Inclusion criteria required: (a) over 18 years, (b) a minimum of 1 year of hospital stay and (c) voluntary interest in participating in the study. The exclusion criteria were the following: (a) workers who had been in the hospital for less than 1 year, (b) who were doing their university internships, (c) had medical disability, were on leave or vacation period. For these above reasons, the participants were selected from a non-probabilistic convenience sampling. Figure 1 shows the flow of participants.

The participants came from urban (*n* = 29) and rural (*n* = 4) areas of the municipality. Most had only one job (*n* = 30), while three participants had two jobs. The majority had one or more children (*n* = 21). For most participants, this was their first experience with a mindfulness practice (*n* = 29). As shown in Table 1, the average age of the participants was 35.03, most were women (*n* = 26), and the predominant marital status was civil union (*n* = 16). In addition, most of the participants had technical and professional training (*n* = 26) and performed care (*n* = 15) and administrative (*n* = 18) roles. The number of randomized participants (*N* = 35) was determined through a statistical power analysis (*α* = 0.05, *β* = 0.20; Faul et al., 2007).

**Table 1:** Sociodemographic characteristics of participants.

	(*n* = 33)	(*n* = 16)	(*n* = 17)	
Variable	Total	Experimental	Control	*p*
Age, *M* (SD)	35.03 (10.00)	34.00 (9.59)	36.03 (10.56)	0.574
Gender				
Female, *n* (%)	26 (78.8)	14 (87.5)	12 (70.6)	0.235
Male, *n* (%)	7 (21.2)	2 (12.5)	5 (29.4)	
Marital status				
Married, *n* (%)	4 (12.1)	2 (12.5)	2 (11.8)	0.808
Single, *n* (%)	12 (36.4)	6 (37.5)	6 (35.3)	
Divorced, *n* (%)	1 (3.03)	0 (0.0)	1 (5.8)	
Civil union, *n* (%)	16 (48.5)	8 (50.0)	8 (47.05)	
Schooling				
Primary school, *n* (%)	1 (3.03)	0 (0.0)	1 (5.8)	0.104
High School, *n* (%)	2 (6.06)	1 (6.3)	1 (5.8)	
Technician, *n* (%)	13 (39.3)	3 (18.7)	10 (58.8)	
Undergraduate, *n* (%)	13 (39.3)	9 (56.2)	4 (23.5)	
Graduate, *n* (%)	4 (12.1)	3 (18.7)	1 (5.8)	
Role				
Healthcare, *n* (%)	15 (45.5)	5 (31.3)	10 (58.8)	0.112
Administrative, *n* (%)	18 (54.5)	11 (68.7)	7 (41.2)	

### 2.2 Instruments

Sociodemographic and labor datasheet: this *ad hoc* questionnaire prepared by the researchers asked about age, gender, marital status, level of schooling, and role (healthcare/administrative).

Five Facets of Mindfulness Questionnaire (FFMQ): an instrument made up of 39 items covering five facets: observing, describing, acting with awareness, non-judging, and non-reacting. It is answered using a five-point Likert-type scale ranging from 0 = never to 5 = very often. An example of an item is: “I perceive my feelings and emotions without having to react to them.” It has a reverse rating for 19 items. The FFMQ factors indicated adequate reliability values through coefficient alpha (observing, *α* = 0.81; describing, *α* = 0.91; acting with awareness, *α* = 0.89; non-judging *α* = 0.91; non-reacting, *α* = 0.80). This instrument also demonstrated construct validity for the model of five intercorrelated factors with satisfactory fit indices (Cebolla et al., 2012). In Colombia, the study of its psychometric properties confirms the 5-factor structure (Sosa and Bianchi, 2019).

Psychological capital in organizations (Ipsicap-24): An instrument composed of 24 items accounting for the following variables: hope, optimism, resilience, and self-efficacy. It uses a 6-point Likert-type scale ranging from 1 = completely disagree to 6 = completely agree. An example of an item is: “I identify strategies to achieve my goals.” The Ipsicap-24 factors indicated reliability values through the alpha coefficient (hope, *α* = 0.84; optimism, *α* = 0.78; resilience, *α* = 0.83; self-efficacy, *α* = 0.71). The validation form for the Colombian population was used (Delgado-Abella and Mañas, 2019).

The Professional Quality of Life Questionnaire (ProQOL -IV) consists of 30 items assessing three dimensions: secondary traumatic stress, burnout, and compassion satisfaction. It is answered on a Likert scale from 0 = never to 5 = always. An example of an item is: “I am happy.” The ProQOL factors reported adequate internal consistency through coefficient alpha (compassion satisfaction, *α* = 0.81; secondary traumatic stress, *α* = 0.84; burnout, *α* = 0.77) (Lago and Codo, 2013). The Colombian adaptation form was used in this study (Piragauta, 2022).

Positive and Negative Affect Schedule (PANAS): this scale evaluates positive and negative affect based on 20 items (10 for positive and 10 for negative affect). Its Likert-type response format has five options, from 1 = never to 5 = always. An example of an item is: “Excited.” A previous study involving a Colombian sample confirmed the original factorial model and indicated adequate reliability through the omega coefficient (positive affect, *ω* = 0.87; negative affect, *ω* = 0.85) (Moreta-Herrera et al., 2021).

### 2.3 Procedure

First, a meeting was held with the hospital manager to present the study. An instructive poster was used to inform the workers about the study’s objective. Next, the institution’s manager and psychologist extended the invitation to participate in the research in different meetings. The participants enrolled, and then they were randomly assigned to the wait-list control group and the experimental group. The people who were part of the experimental group received training in MBHP. Once the experiment was completed, the general results were socialized in the institution’s steering committee.

#### 2.3.1 Mindfulness-based health promotion (MBHP)

MBPH is the program designed by the Mente Aberta Center, which is inspired by the Mindfulness-Based Stress Reduction, Mindfulness-Based Cognitive Therapy, and Mindfulness-Based Relapse Prevention models (Duran et al., 2022).

It is a structured program of 8 sessions. This protocol is directed to the Hispanic/Latin American context and emphasizes four fundamental practices: breathing, walking, body scanning, and movements. In addition, the program proposes exercises based on Buddhist practices of compassion and self-compassion. Participants also receive suggestions for daily activities to implement at home or in the workplace, which last 15–20 min on average (Demarzo, 2020; Duran et al., 2022).

For the purposes of the experiment, the training lasted 2 months, with sessions of 2 h per week, which were scheduled during the participants’ workday. It was carried out in the Casa de la Cultura facilities where the hospital is located. It was led by a psychologist certified as an instructor in Mindfulness na Promoção da Saude by the Universidade Federal do Estado de São Paulo (UNIFESP) and by the Centro Brasileiro Mente Aberta.

The sessions with the experimental group focused on: (1) What is Mindfulness? Getting out of autopilot; (2) The Mindfulness of breathing; (3) Mindfulness in daily life; (4) Mindfulness for challenging situations; (5) Mindfulness of mind and thoughts; (6) Silence; (7) Mindfulness and compassion, and (8) Mindfulness for life. Each session was accompanied by active methodologies such as experiential activities, breathing exercises, conscious eating, group activities, metaphors, videos, conscious dialogs, conscious walking, and body scan. In addition, each participant was given a mindfulness diary where they reported their progress and experiences with the assigned tasks weekly.

#### 2.3.2 Wait-list control group

The wait-list control group only received a lecture on the conceptualization of mindfulness and its characteristics during the study. For ethical reasons, once the intervention was completed with the experimental group and the post-test was carried out with both groups, the wait-list control group received three mindfulness practice sessions, held weekly for 1 h and 30 min. These sessions covered topics such as breathing, stepping out of autopilot, compassion, observing thoughts, and mindfulness in challenging situations.

### 2.4 Statistical analysis

The experiment was subjected to the following statistical treatment: an exploratory data analysis was performed based on the technical recommendations (Hair et al., 2014) to estimate measures of central tendency (M), dispersion (SD), and Confidence Interval (95% of the mean). No outliers were identified by visual scanning (Boxplot) or the interquartile range (Q3-Q1). In these cases, each piece of data was analyzed by determining the information collection process. In some cases, the Winsorizing technique was applied to adjust the psychological measures.

Each analysis of the experiment was performed separately to estimate and control the effect of each psychological variable. Shapiro Wilk normality assumptions (*n* < 50) were checked for the four models. The comparison of demographic and contextual variables was estimated with the chi-square and t-student test for independent samples. The contrast of the effects of the Mindfulness Program for health promotion was performed through the mixed factorial ANOVA or partially repeated measures. The dependent variables were treated separately for their analysis conditions containing each of these measures (e.g., dimensions per psychological construct).

Four factorial models were estimated:

- The first model was 2×2 for Mindfulness: six dependent variables associated with mindfulness (observing, describing, acting with awareness, non-judging, non-reacting, mindfulness) × 2 time (pre-test/post-test) × 2 groups (experimental and control).
- The second model was 2×2 for psychological capital: five dependent variables associated with psychological capital (hope, optimism, resilience, self-efficacy, psychological capital) × 2 time (pre-test/post-test) × 2 groups (experimental and control).
- The third model was 2×2 for compassion fatigue: four dependent variables associated with compassion fatigue (secondary traumatic stress, burnout, compassion satisfaction, compassion fatigue) × 2 time (pre-test/post-test) × 2 groups (experimental and control).
- The fourth model was 2×2 for affect: two dependent variables associated with affect (positive affect, negative affect) × 2 time (pre-test/post-test) × 2 groups (experimental and control).

The significance of interactions and main effects were analyzed with the Bonferroni post-hoc test. The effect size was estimated with partial eta squared. Finally, statistical treatment was performed with the JASP software version 0.9.

### 2.5 Ethical considerations

We adhered to the Declaration of Helsinki (World Medical Association, 2008), the Universal Declaration of Ethic Principles for Psychologist regulations (International Union of Psychological Science, 2008), and the guidelines of Law 1090 of 2006 (Congreso de la República de Colombia, 2006). All participants signed the informed consent form, where the purpose of the experiment, the risks, and the intervention protocol were informed. The research was approved by the Ethics Committee of the University that led the project (Act no. 05).

## 3 Results

After the randomization of the groups, out of 100% of the participants included (*N* = 35), the dropout rate was 6 and 94% completed the intervention. The descriptive statistics (mean and standard deviation) of the participants in the experimental and wait-list control groups are shown in Table 2.

**Table 2:** Descriptive data of the experimental and wait-list control groups according to the (Pre-test/Post-test) measurement.

Variable	Experimental (*n* = 16)				Control (*n* = 17)			
	Pre-test		Post-test		Pre-test		Post-test	
	*M*	SD	*M*	SD	*M*	SD	*M*	SD
Observing	25.6	5.5	32.3	5.4	24.2	6.1	24.2	6.3
Describing	17.8	4.1	18.9	5.0	18.1	3.4	17.0	3.7
Acting with Awareness	29.8	5.6	28.9	4.2	29.8	5.3	26.4	7.8
Non-Judging	28.6	6.5	28.6	5.8	28.4	4.9	25.2	6.6
Non-Reacting	35.6	5.0	37.8	4.2	30.4	5.1	32.5	5.2
Mindfulness	137.3	19.2	146.4	16.1	131.0	15.4	125.3	20.7
Hope	35.2	6.3	38.3	3.5	35.4	6.4	34.2	7.0
Optimism	34.3	5.6	36.0	3.2	33.8	4.4	34.5	5.2
Resilience	29.5	4.5	31.6	2.9	29.4	5.2	28.9	4.8
Self-Efficacy	18.7	2.6	19.9	1.9	19.0	2.0	18.4	3.1
Psychological Capital	117.6	17.3	125.7	8.6	117.6	13.9	115.9	18.1
Secondary Traumatic Stress	21.9	9.1	18.3	6.7	21.5	11.1	23.1	9.5
Burnout	21.0	7.0	17.3	5.1	17.5	5.8	19.5	6.0
Compassion Satisfaction	42.4	5.0	44.4	4.3	45.3	3.2	44.1	3.5
Compassion Fatigue	28.4	5.4	26.7	3.5	28.0	5.3	29.0	4.7
Positive Affect	40.8	5.5	42.5	4.7	40.4	6.4	42.6	3.4
Negative Affect	20.3	5.8	19.2	5.8	19.1	6.1	20.2	5.0

M, Mean; SD, Standard Deviation.

### 3.1 The mix ANOVA 2×2 for mindfulness

**Figure 2:**
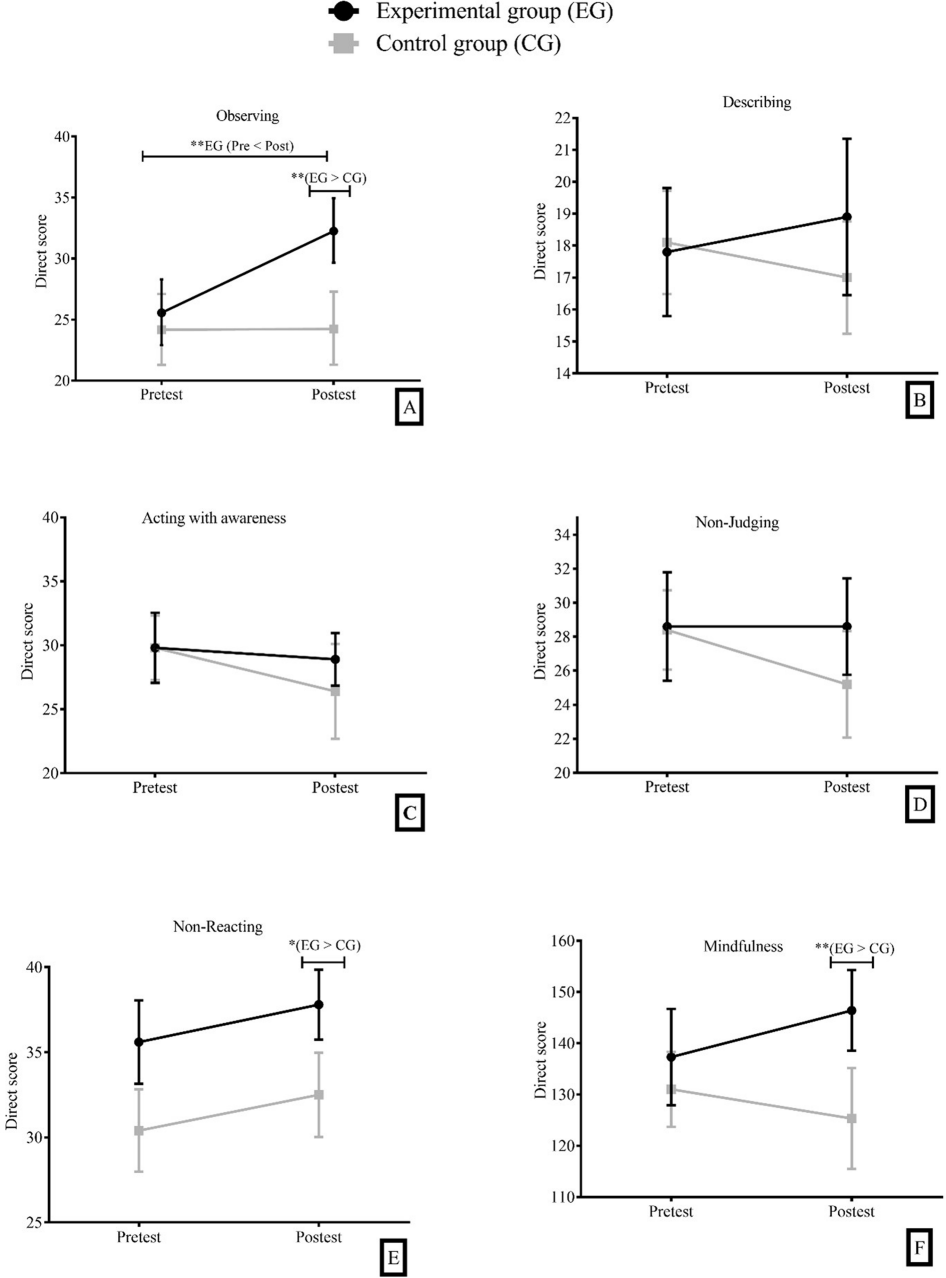
**(A)** Observing; **(B)** Describing; **(C)** Acting with awareness; **(D)** Non-Judging; **(E)** Non-Reacting; **(F)** Mindfulness. Means and 95% confidence intervals in the six mindfulness variables, (pre-test and post-test) time factor, (Experimental Group=EG and Control Group=CG) group factor.

Bonferroni *post hoc* analysis indicated significant differences between the experimental group and the wait-list control in the post-test on the variables observing (M*_diff_* = 8.0, CI 95% [2.45, 13.58], *p* < 0.01, *η^2^_p_* = 0.19), no-reacting (M*_diff_* = 5.34, CI 95% [0.69, 10.0], *p* < 0.05, *η^2^_p_* = 0.35) and mindfulness (M*_diff_* = 21.1, CI 95% [3.93, 38.4], *p* < 0.01, *η^2^_p_* = 0.17; Figure 2). However, no significant results were obtained between the experimental group and the wait-list group at posttest on the other variables: describing (M*_diff_* = 1.87, CI 95% [−2.03, 5.78], *p* = 0.12), acting with awareness (M*_diff_* = 2.53, CI 95% [−3.11, 8.16], *p* = 0.29) and non-judging (M*_diff_* = 3.39, CI 95% [−2.33, 9.10], *p* = 0.66).

### 3.2 The mix ANOVA 2×2 for psychological capital

**Figure 3:**
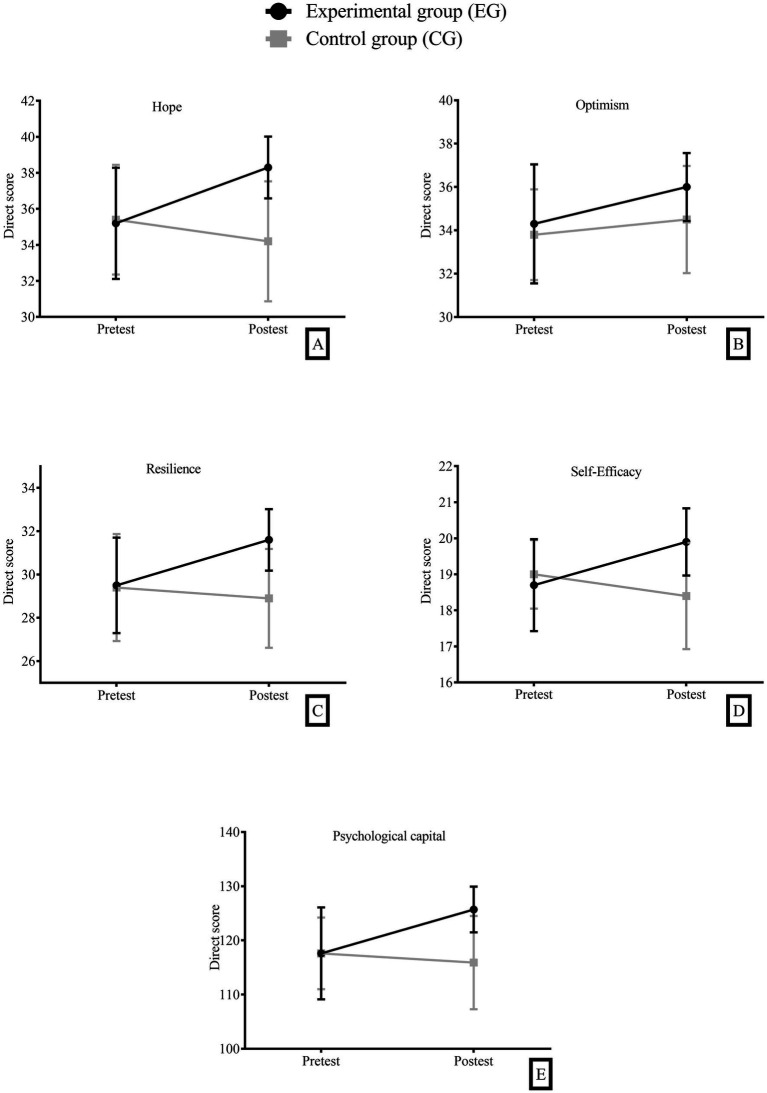
**(A)** Hope; **(B)** Optimism; **(C)** Resilience; **(D)** Self-Efficacy; **(E)** Psychological capital. Means and 95% confidence intervals in the five psychological capital variables, (Pre-test and Post-test) time factor, (Experimental Group=EG and Control Group=CG) group factor.

The mean score of the participants in the experimental group on the post-test time was higher than that of the wait-list control group. On the other hand, the interaction between the variables associated with psychological capital, time and group was not significant: hope (M*_diff_* = 4.07, CI 95% [−2.33, 9.10], *p* = 0.34), optimism (M*_diff_* = 1.53, CI 95% [−2.97, 6.03], *p* = 0.51), resilience (M*_diff_* = 2.62, CI 95% [−1.66, 6.91], *p* = 0.59), self-efficacy (M*_diff_* = 1.52, CI 95% [−0.82, 3.86], *p* = 0.49), psychological capital (M*_diff_* = 9.75, CI 95% [−4.55, 24.05], *p* = 0.40) (Figure 3).

### 3.3 The mix ANOVA 2×2 for compassion fatigue

**Figure 4:**
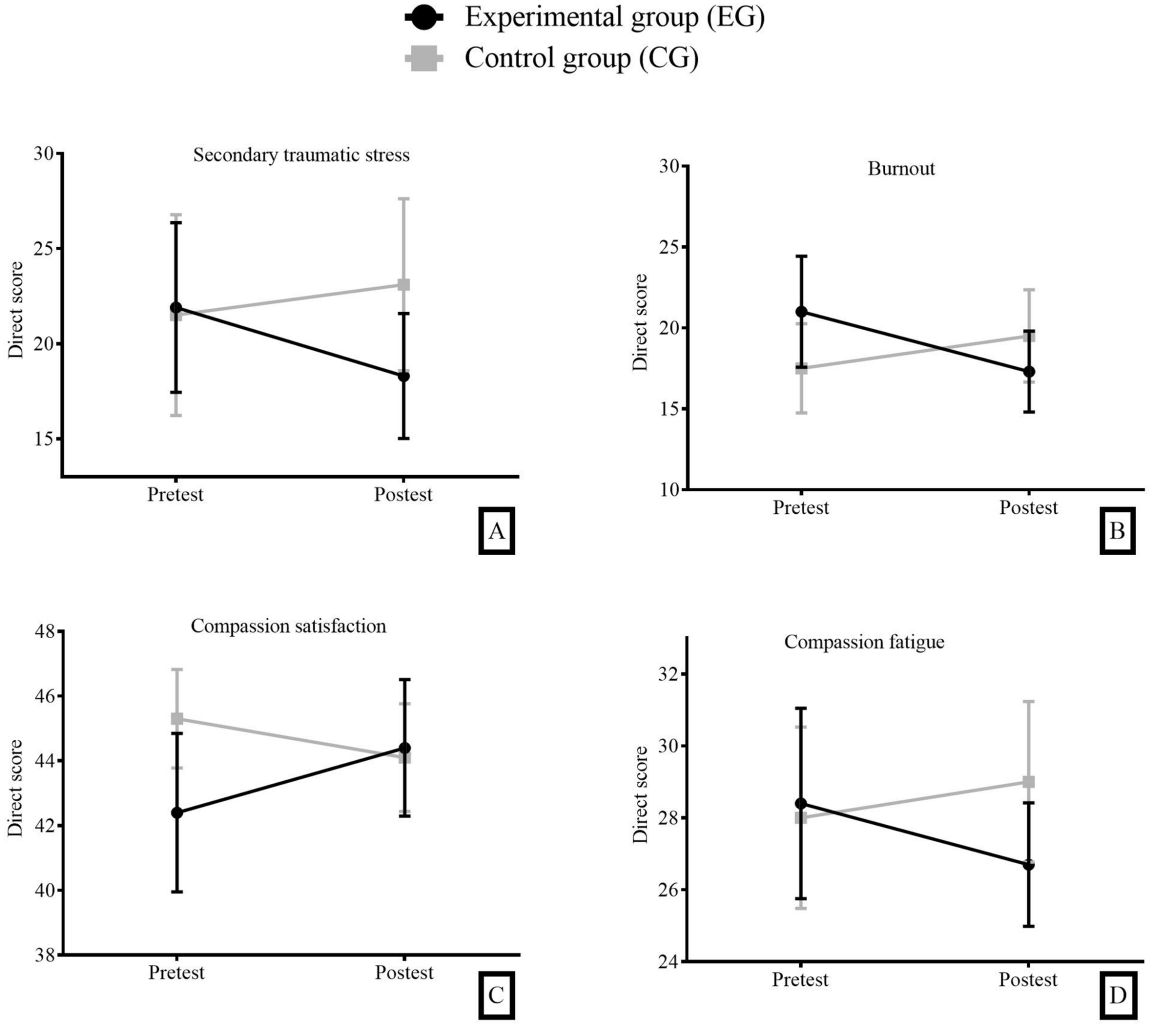
**(A)** Secondary traumatic stress; **(B)** Burnout; **(C)** Compassion satisfaction; **(D)** Compassion fatigue. Means and 95% confidence intervals in the four compassion fatigue variables, (Pre-test and Post-test) time factor, (Experimental Group - EG and Control Group - CG) group factor.

In the *post hoc* analysis, the variables associated with compassion fatigue: secondary traumatic stress (M*_diff_* = −4.81, CI 95% [−13.70, 4.09], *p* = 0.86), burnout (M*_diff_* = −2.22, CI 95% [−7.94, 3.50], *p* > 0.05), compassion satisfaction (M*_diff_* = 0.26, CI 95% [−3.60, 4.12], *p* = 0.29) and compassion fatigue (M*_diff_* = −2.31, CI 95% [1.67, −1.38], *p* = 0.13), were not significant between the experimental group and the wait-list control in the post-test (Figure 4).

### 3.4 The mix ANOVA 2×2 for affect

Finally, no significant results were obtained between the experimental group and the wait-list group at posttest on the variables: positive affect (M*_diff_* = −0.15, CI 95% [−5.04, 4.75], *p* = 0.74) and negative affect (M*_diff_* = −0.99, CI 95% [−6.43, 4.45], *p* = 0.27).

## 4 Discussion

This research explored the effects of MBHP on mindfulness, psychological capital, compassion fatigue, and positive/negative affect in a sample of healthcare and administrative staff at a hospital in Colombia. Our findings add to the evidence suggesting that mindfulness programs in the work environment can be an alternative to promote well-being in workers, specifically health professionals (Bartlett et al., 2019; Lomas et al., 2019).

One of the innovative contributions of this study is the use of a novel mindfulness-based intervention (MBHP) designed for a Hispanic/Latin American audience. The fact that it was used with healthcare workers contributes to broadening the spectrum of research on MBHP carried out with other population groups, such as police officers (Trombka et al., 2021), teachers (Rodrigues de Oliveira et al., 2021) or patients (Duran et al., 2022).

From the analyses conducted, only the first of the four tested factorial models could be confirmed. Based on the background information consulted, it was hypothesized that a more significant effect would be obtained by considering the overall score of the FFMQ rather than the scores of the dimensions making up the test. From the results obtained, a significant difference was found in the total mindfulness score and two dimensions, with higher scores in the experimental group in the post-test measure compared to the wait-list control group. This confirms the first hypothesis (H1) proposed. The results align with previous research that has shown mindfulness practice is associated with increased trait mindfulness (Van Dam et al., 2009; Goldberg et al., 2016; Quaglia et al., 2016).

In addition to the differences in the total mindfulness score, significant differences were found in two of the FFMQ dimensions. On the one hand, participants who engaged in mindfulness practice reported higher values in the Non-reacting dimension. This finding is consistent with the consulted literature, which often reports a more significant effect on this dimension (Rimes and Wingrove, 2011; Asuero et al., 2014). This result indicates that participating in MBHP sessions helped participants acquire strategies to better manage emotions and thoughts, avoiding impulsive responses. This is crucial for healthcare workers who are exposed to stressful situations daily. On the other hand, the second dimension that showed significant results was Observing. This finding highlights the effectiveness of the MBHP protocol in promoting greater attention to sensations, emotions, and thoughts in the participants. Activities such as body scanning, mindful walking, mindful eating, and breathing exercises appear to be essential in achieving these gains.

The second hypothesis (H2) formulated was not verified because MBHP training showed a tendency to increase in the experimental group for measures of hope, optimism, resilience, self-efficacy, and psychological capital, but this was not significant. Although several previous studies have reported that different types of mindfulness interventions have generated improvements in the levels of psychological capital (Jain and Singh, 2016; Xu et al., 2021; Biswal and Srivastava, 2022), this did not occur in this research.

From the Resource Conservation Theory (RCT), the result obtained with psychological capital in this study could be discussed since it provides a framework for understanding, predicting, and studying the processes of interaction between people and the contexts in which they operate. In the context of RCT, there are fertile or infertile environments for the creation, maintenance, and limitation of resources. Put differently, the existence and conservation of resources depends on certain ecological, social, and environmental conditions (Hobfoll et al., 2018). Specifically, the four components of psychological capital are considered positive personal resources (Luthans et al., 2010; Warren et al., 2023) that require minimum organizational and environmental conditions to grow (Hobfoll, 2011), and the circumstances in a healthcare service, given the continuous stress to which personnel are exposed, might not have been ideal for enhancing psychological capital.

Hypothesis 3 (H3) proposed a change in compassion fatigue. Based on the results obtained, this hypothesis could not be confirmed either. Although the systematic review conducted by Conversano et al. (2020) reports that other types of mindfulness training have shown changes in levels of compassion fatigue (and its dimensions) after training, in the case of MBHP, this could not be proven. However, it is important to note that in their review study, Conversano et al. (2020) report controversial findings on this topic, associated with differences in gender and years of work experience. Additionally, if we consider some dimensions of the construct, such as burnout, some studies show mixed results. For instance, Biswal and Srivastava (2022) worked with a sample of leaders and reported non-significant differences in burnout after MBI training. Therefore, it is possible that if MBHP training were conducted with a larger sample of healthcare workers, allowing for the analysis of differences based on gender, years of experience, and type of position, the results might indicate differences in favor of some of these sub-groups.

The fourth hypothesis (H4) postulated that participants going through MBHP training would experience changes in measures of positive and negative affect, specifically a decrease in negative affect and an increase in positive affect were expected. No significant effects were obtained for either positive or negative affect. It can be said that this hypothesis was not fulfilled as expected. Consistent with these findings, experimental studies using protocols other than MBHP have also reported no significant differences between groups regarding positive or negative affect (Jha et al., 2020). However, some studies have shown a significant reduction in negative affect, but not a corresponding significant increase in positive affect (Keng et al., 2021; Sousa et al., 2021). A longitudinal study found that total mindfulness scores predicted a decrease in negative affect over 3 months, but no improvement in positive affect was observed (Jose and Geiserman, 2024).

In summary and as a conclusion of the study conducted, although it could not be confirmed that MBHP had an effect on psychological capital, compassion fatigue, and affect in a sample of healthcare workers, an effect on mindfulness and its dimensions was confirmed. This indicates that the program is effective in developing awareness, that is, being conscious of internal and external stimuli. This allows for more assertive responses and reacting less impulsively to situations, thereby increasing self-efficacy and quality of life (Duran et al., 2022). Consistent with the above, the job demands-resources theory (Bakker et al., 2023; Demerouti and Bakker, 2023) helps explain this finding from this study. Grover et al. (2017) suggests that mindfulness acts as a protective resource that can mitigate work demands. As a personal resource, mindfulness positively influences the work experience by reducing stress and buffering perceptions of job demands. Similar results have been reported in other studies (Janssen et al., 2020; de Carvalho et al., 2021).

It is important to highlight the limited research conducted in Latin American countries that examines the effects of mindfulness practices on healthcare workers (Juárez García et al., 2022). In this sense, our findings have important theorical and practical implications. Among the strengths of this study is the use of a novel mindfulness program (MBHP) specifically designed for healthcare workers in the Latin American context. These results contribute to the challenging task of promoting the mental health of those who care for the population’s health. The relevance and social impact of these findings provide valuable input for leaders of healthcare institutions to prioritize evidence-based interventions aimed at safeguarding the mental health of healthcare workers.

Notably, in Colombia human resource management has been increasingly positioning itself as a strategic field for achieving business objectives (Calderón-Hernández et al., 2023). As a result, leaders of healthcare institutions should advocate for the adoption of evidence-based intervention programs, such as the MBHP.

### 4.1 Limitations and future research directions

The empirical results reported here have several limitations that need to be discussed. On the one hand, our study only analyzed the effects of the program in those participants who completed all the sessions. However, no measure was used to evaluate adherence to the program. For example, the number of sessions completed by individuals who did not finish the intervention was not recorded, nor was there a log for each participant regarding the completion of the tasks assigned in each session. According to Winter et al. (2022), adherence is not sufficiently reported in research, which is why stricter measurements of adherence are required in future studies to fully understand the role of adherence in the success of interventions. According to some reviews (Dziego et al., 2024), certain personal variables (e.g., personality traits) may influence adherence to treatments. Therefore, it would be useful for future research to analyze these aspects, not only to inform the results of the studies but also to predict long-term adherence of individuals with similar characteristics to mindfulness programs outside of the intervention (Beintner et al., 2019; Ribeiro et al., 2018).

On the other hand, our sample size is small because the participating hospital is a level I complexity entity located in a municipality in Colombia. Furthermore, the funding of this project did not allow mindfulness training in different locations simultaneously. The above makes it difficult to have the best statistical estimators for the control and experimental groups; the impacts of a small random sample have been described in specialized literature (Lawson, 2015). In addition, the absence of cultural validity and the indirect measurement of our questionnaires propitiate measurement error that was not controlled, detailed threats in experimental designs applied in psychology. Thus, our findings should be interpreted carefully and critically. Likewise, having larger sample sizes, especially in experimental studies, is also part of the co-responsibility of the Institutions that participate in the investigative processes, since it is important to monitor the permanence in intervention practices and favor permits so that workers can assisted.

Future research could include a more significant number of participants and medium- and long-term follow-ups. More funding would allow biomarkers to be included in the study to contrast psychological and physiological measures in healthcare workers. Finally, future research could have mixed designs, which would expand the understanding of the phenomena studied here.

## 5 Conclusion

This study contributes to the growing research on mindfulness programs for health care workers and is pioneering in using MBHP in this occupational group in Colombia. Our findings indicate that this mindfulness program has the potential to improve mindfulness and its dimensions of observing and non-reacting in this type of workers. The relevance and social impact of these findings are an input for decision makers in organizations to prioritize evidence-based interventions.

## Data Availability

The datasets presented in this study can be found in online repositories. The names of the repository/repositories and accession number(s) can be found below: https://osf.io/sn3hf/?view_only=65f5633ab2cb46deb7ac5ef72826c6ac.
